# A chromosome-scale genome assembly and annotation of the tetraploid herb “epazote” (*Dysphania ambrosioides*)

**DOI:** 10.1093/g3journal/jkaf191

**Published:** 2025-08-19

**Authors:** Paul B Frandsen, Abigail Borgmeier, Sam Bratsman, Brian J Cox, Sarah J Gottfredson, Robert Hadfield, Garrett Harding, Andrea L Kokkonen, Ying Fei Lin, Jackson Linde, Teagan Mulford, Andrew Parker, Shane Smith, Kaitlin Torres, Lauren Young, Hayley Mangelson, Eric N Jellen, Peter J Maughan, David E Jarvis

**Affiliations:** Department of Plant and Wildlife Sciences, Brigham Young University, Provo, UT 84602, United States; Department of Biology, Brigham Young University, Provo, UT 84602, United States; Department of Plant and Wildlife Sciences, Brigham Young University, Provo, UT 84602, United States; Department of Plant and Wildlife Sciences, Brigham Young University, Provo, UT 84602, United States; Department of Plant and Wildlife Sciences, Brigham Young University, Provo, UT 84602, United States; Department of Biology, Brigham Young University, Provo, UT 84602, United States; Department of Plant and Wildlife Sciences, Brigham Young University, Provo, UT 84602, United States; Department of Biology, Brigham Young University, Provo, UT 84602, United States; Department of Plant and Wildlife Sciences, Brigham Young University, Provo, UT 84602, United States; Department of Biology, Brigham Young University, Provo, UT 84602, United States; Department of Biology, Brigham Young University, Provo, UT 84602, United States; Department of Plant and Wildlife Sciences, Brigham Young University, Provo, UT 84602, United States; Department of Plant and Wildlife Sciences, Brigham Young University, Provo, UT 84602, United States; Department of Plant and Wildlife Sciences, Brigham Young University, Provo, UT 84602, United States; Department of Plant and Wildlife Sciences, Brigham Young University, Provo, UT 84602, United States; Phase Genomics, Seattle, WA 98109, United States; Department of Plant and Wildlife Sciences, Brigham Young University, Provo, UT 84602, United States; Department of Plant and Wildlife Sciences, Brigham Young University, Provo, UT 84602, United States; Department of Plant and Wildlife Sciences, Brigham Young University, Provo, UT 84602, United States

**Keywords:** plants, epazote, genome assembly, genome annotation, *Dysphania ambrosioides*

## Abstract

Epazote (*Dysphania ambrosioides* L.) is a perennial plant from the tropics of the Americas and is of regional importance due to both culinary and medicinal applications. However, few genomic resources exist to facilitate the identification of genes and pathways underlying the production of functionally important compounds in epazote. Here, we present a chromosome-scale assembly of the tetraploid epazote genome using PacBio HiFi reads and Hi-C. The final genome assembly contains 191 scaffolds spanning a total length of 469.23 Mbp, with 98.17% of the total length in the 16 largest chromosome-scale scaffolds. A BUSCO analysis identified 99.01% of the universal, single-copy orthologs, indicating that the genome is largely complete. We identified 51.81% of the genome as repetitive and annotated 24,424 genes. Collinearity of homologous genes has degraded to the point that, with few exceptions, homoeologous chromosome pairs cannot be identified, suggesting that the whole-genome duplication in epazote is relatively old. Analysis of epazote and related species suggests that the whole-genome duplication in epazote is independent and is older than the whole-genome duplication in quinoa but younger than that of amaranth.

## Introduction

Epazote (*Dysphania ambrosioides* L.) is a polymorphic annual or short-lived perennial plant native to the tropical regions of the Americas (Mosyakin and Clemants 2008) and is commonly used as an anthelmintic and antifungal agent as well as an herb in food preparation (Gadano et al. 2002). Originally classified as *Chenopodium ambrosioides* in the family Chenopodiaceae, the species was transferred to *D. ambrosioides* within the Amaranthaceae family (Kadereit et al. 2003; Müller and Borsch 2005; Fuentes-Bazan, Mansion, et al. 2012a). Most *Dysphania* species have been found to have a base chromosome number of *x* = 8, although some *x* = 9 species have been described, and ploidy levels range from diploid to octoploid (Grozeva and Cvetanova 2013; Uotila et al. 2021). Epazote is a tetraploid with a base chromosome number of 8 (2*n* = 4*x* = 32).

Epazote has been introduced around the world and can now be found along roadsides, waste areas, and riparian habitats on all continents and many oceanic islands. Due to its ubiquity, epazote is known by several other common names, including Mexican tea, Jesuit's tea, American wormseed, paico (or paycu), mastruz, apazote, and erva-de-Santa-Maria (Gadano et al. 2002; Sá et al. 2016).

Epazote can reach a height up to 1 m and is highly branched. Its leaves are alternate, elongated, hairy, and jagged, exhibiting various shades of green (Kumar et al. 2007). Leaf size varies, with the smallest leaves positioned at the plant's apex and the largest at its base. Its inflorescence is unbranched with small green flowers and numerous black, spherical seeds with a flaky pericarp (Gadano et al. 2002; Sá et al. 2016). On their epidermal layer, epazote leaves have specialized tissues that store and secrete secondary metabolites (Samanani and Facchini 2006).

Past research on epazote has focused primarily on the production of secondary metabolites, emphasizing the historic uses of its oil extract and leaves. Epazote extract has been used for various medicinal and agricultural applications, although only a few specific compounds have been matched to a function. The epazote extract ascaridol has drawn attention as a viable anthelmintic and antitumor agent (Nascimento et al. 2006) but has proven fatal in large doses (Pereira et al. 2010).

While there are several molecular studies focusing on the tribe Dysphanieae (Fuentes-Bazan, Mansion, et al. 2012a; Sukhorukov et al. 2018; Uotila et al. 2021) and many more on the family Amaranthaceae (Coles et al. 2005; Bhargava et al. 2006; Christensen et al. 2007; Kolano et al. 2016; Mandák et al. 2016), few studies have looked specifically at the genus *Dysphania* (Kim et al. 2019 ; Park and Kim 2019), the majority of which only focus on 1 species, the invasive weed *Dysphania pumilio*. Currently, there are no studies using epazote whole-genome sequencing data to infer evolutionary relationships or to further understand this group of plants. Genomic data are vital for determining and resolving the evolutionary relationships within families and are considered a more reliable approach than morphology due to convergence (Wake et al. 2011; Legg et al. 2013; Springer et al. 2013; Jarvis et al. 2014; Zou and Zhang 2016). Since epazote is part of a group of plants that have a complicated taxonomic history (Fuentes-Bazan, Uotila, et al. 2012b) due, in part, to varying interpretations of morphological characteristics, genomic data could help resolve this history. Genomic studies could also provide useful information on plant secondary metabolism, including plant domestication and the evolution of secondary metabolic pathways (Zhao et al. 2020). Understanding the genomic components and molecular mechanisms underlying secondary metabolite production will facilitate the future application of these metabolites in medicine and other beneficial avenues.

Here, we report a high-quality annotated genome for epazote. This genome resource can be used to further clarify the evolutionary history of epazote and aid in identifying and characterizing secondary metabolic pathways.

## Methods

### Genome sequencing and size estimation

The epazote reference genome assembly and annotation were generated using *D. ambrosioides* accession PI 604781, using seed obtained from the USDA-ARS Germplasm Resources Information Network (GRIN, https://www.ars-grin.gov/). Leaf tissue from a single plant was sampled, extracted, and fragmented at the BYU DNA Sequencing Center using the Circulomics Nanobind DNA extraction kit. The BYU DNA Sequencing Center selected for 15-kb fragments using a Sage Science BluePippin, and library prep was conducted using the PacBio SMRTbell library kit. The sequencing was carried out using PacBio circular consensus sequencing (ccs) technology on the PacBio Sequel II instrument. HiFi reads (i.e. reads with quality score > Q20) were generated from the ccs reads using the PacBio SMRT analysis software. Genome size was estimated from all the HiFi reads with Jellyfish v2.2 (Marçais and Kingsford 2011) using a *k*-mer size of 21 and an initial hash of 10 million and the web version of GenomeScope 2.0 (http://genomescope.org/genomescope2.0/) (Ranallo-Benavidez et al. 2020) using a max *k*-mer coverage of 1,000,000.

### Genome assembly

HiFi reads with lengths greater than 12 kbp were selected using fastp (Chen et al. 2018) and assembled into contigs using the default settings of hifiasm v0.7 (Cheng et al. 2021, 2022, 2024). Hi-C library prep, sequencing, and scaffolding were performed by Phase Genomics, using leaf tissue.

### Assembly validation

Identification of putative contaminant sequences was performed with Kraken v2.1.2 (Wood et al. 2019) using the PlusPFP database and with BlobTools (Laetsch and Blaxter 2017). Assembly completeness was assessed with BUSCO v5.2.2 (Manni et al. 2021) using the embryophyta_odb10 database.

### Chloroplast and mitochondria genome assembly

The epazote chloroplast and mitochondrial genomes were assembled and annotated as previously described (Jarvis et al. 2022), using the *Chenopodium quinoa* chloroplast (GenBank accession MK159176.1) and *Beta vulgaris* mitochondrial genome (GenBank accession BA000024.1) as baits, respectively. Any sequence in the genome assembly that displayed greater than 99% identity to the assembled chloroplast or mitochondria over more than 99% of the sequence length was removed from the assembly.

### Genome annotation

Repetitive sequences were identified, classified, and masked using RepeatModeler v2.0.1 (Flynn et al. 2020) and RepeatMasker v4.1.2 (Smit et al. 2013). Telomeric repeats were identified using tidk v0.2.63 (Brown et al. 2025) with the clade option set to Caryophyllales. Genes were identified and annotated using Braker v3.0.7 (Gabriel et al. 2024) as previously described (Jaggi et al. 2025). Expression evidence was provided by PacBio Iso-Seq HiFi reads generated from a pooled sample of RNA extracted from young leaves, old leaves, meristems, stems, and roots. RNA was extracted using the Zymo Direct-zol RNA MiniPrep Plus w/TriReagent Kit (cat. no. R2071-A) following the manufacturer's instructions, and Iso-Seq library preparation and sequencing were performed at the BYU DNA Sequencing Center. The number of genes and telomeric sequences and the length of all other repetitive sequences were visualized in 500-kb windows along the assembled epazote chromosomes using Circa (https://omgenomics.com/circa).

### Whole-genome duplication and phylogenetics

Homologous gene pairs within the epazote genome were identified by performing a self BLASTp search using the num_descriptions 5, num_alignments 5, and e-value 1e-10 settings. Blocks of collinear gene pairs were identified with MCScanX (Wang et al. 2012 ) and visualized as a dot plot in SynVisio (Bandi and Gutwin 2020).

To assess the epazote whole-genome duplication (WGD) in a phylogenetic context, an orthogroup analysis was constructed with Orthofinder2 v2.5.4 (Emms and Kelly 2019) using from longest protein gene models for epazote, 6 *Amaranthus* species, 9 *Chenopodium* species, *B. vulgaris*, *Atriplex hortensis*, and *Spinacia oleracea*. All 19 species have fully assembled, chromosome-scale, genomes. OrthoFinder2 assigned 96.9% of all genes to orthogroups, with a G50 and O50 of 25 and 6,894, respectfully. A rooted species tree phylogeny was produced using the multiple sequence alignment approach in OrthoFinder2, elicited with the “-M msa” option to produce bootstrap values. WGDs were identified using wgd v.2.0.38 (Chen et al. 2024) by leveraging synteny inference and heuristic peak detection with 95% confidence intervals derived from anchor gene pairs. Synonymous substitution rates (Ks) were calculated between paralogous gene pairs to infer WGDs.

### Resequencing

Whole-genome resequencing was performed for 3 additional accessions of *D. ambrosioides* as well as 5 accessions of *D. graveolens* and 1 accession each of *D. anthelmintica*, *D. botrys*, and *Dysphania cristata*. Seed for all accessions was obtained from the USDA-ARS GRIN. DNA sequencing was performed by Novogene using 150-bp paired-end Illumina sequencing. Reads were trimmed with fastp (Chen et al. 2018) and mapped onto the epazote genome assembly using Minimap2 v2.17 (Li 2018). Mapped reads in SAM format were converted to BAM format, sorted, and indexed using SAMtools v1.9 (Danecek et al. 2021). Read mapping depth was calculated using the flagstat function in SAMtools and the map2cov tool from BlobTools (Laetsch and Blaxter 2017).

## Results and discussion

We isolated high-molecular weight DNA from leaves of a single epazote plant (*D. ambrosioides* PI 604781, 2*n* = 4*x* = 32). PacBio DNA sequencing produced 2,373,343 HiFi reads with a total yield of 36.32 Gbp. A *k*-mer frequency-based analysis of the PacBio HiFi reads using GenomeScope produced a genome size estimate of approximately 425 Mbp (see Supplementary Fig. 1). We selected all HiFi reads > 12 kbp in length for genome assembly, representing 34.61 Gbp of total data, or approximately 82× coverage of the estimated genome size. Contig assembly with hifiasm produced an initial assembly comprised of 476 contigs spanning a total length of 477.12 Mbp (Table 1). The contig N50 was 13.45 Mbp, and the longest contig was 33.55 Mbp.

**Table 1.** Contig and scaffold assembly statistics.

	Contig	Preliminary scaffold	Final
Total length (Mbp)	477.12	477.13	469.23
Total contigs/scaffolds	476	439	191
Longest contig/scaffold (Mbp)	33.55	36.48	36.48
Contig/scaffold N50 (Mbp)	13.45	29.64	29.64
Contig/scaffold L50	12	8	8
Contig/scaffold N90 (Mbp)	4.21	22.92	22.92
Contig/scaffold L90	33	15	15
Gaps	0	37	37

**Fig. 1.**
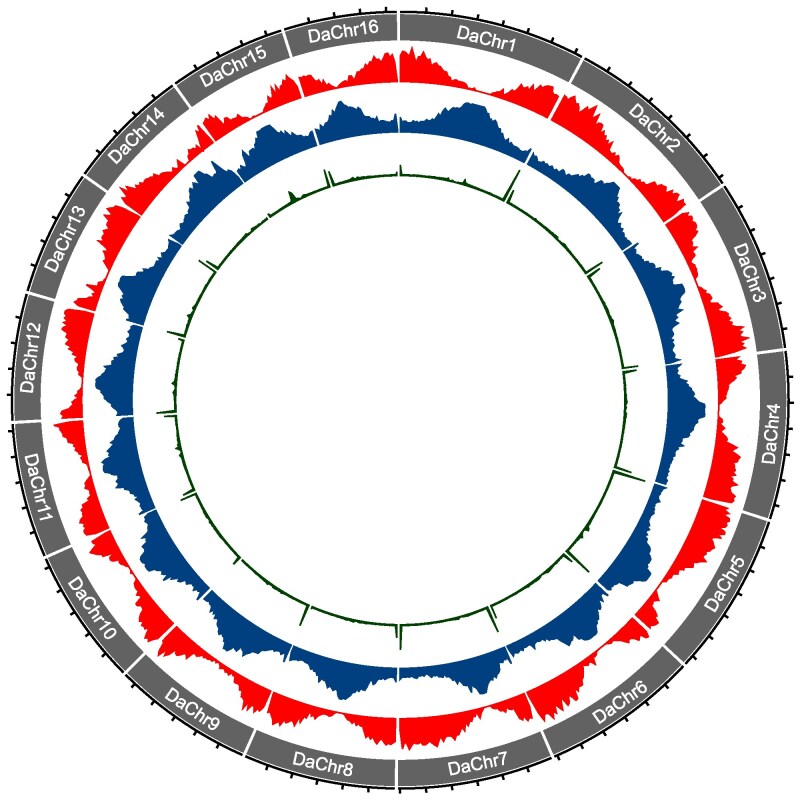
Circular representation of the 16 chromosome-scale scaffolds in the epazote genome assembly. Starting from the outside, tracks represent chromosome size (tick marks, 5 Mbp), chromosome name, gene density, repeat density, and telomeric repeat density.

A Hi-C library was produced by Phase Genomics, and 183,254,025 sequencing read pairs were generated from the library. Scaffolding of the contig assembly with the Hi-C reads produced a preliminary assembly of 439 scaffolds with a scaffold N50 of 29.64 Mbp (Table 1). Sixteen of the scaffolds were substantially larger than all others and contained 460.63 Mbp (96.54%) of the total sequence length, presumably corresponding to the 16 expected haploid chromosomes. We named these scaffolds from largest to smallest as DaChr1–DaChr16 (Fig. 1).

We assessed the preliminary scaffold assembly using Kraken2 and BlobTools to identify potential contaminant sequences. Kraken2 categorized 1 scaffold as *Homo sapiens* and 4 as unclassified taxonomy. Further investigation of these scaffolds revealed that all were short (<100 kb) and comprised of only 1 contig each. A BLAST search of each of these scaffolds against the NCBI nt database obtained no hits longer than 100 bp or with e-values < 1.3; therefore, because these contigs could not be definitively identified as contaminants, they were not removed from the assembly. Likewise, BlobTools did not identify any sequences that were definitively contaminants based on sequencing depth, GC%, or homology (see Supplementary Fig. 2).

We assembled complete chloroplast and mitochondria genomes from the PacBio DNA sequencing reads. The chloroplast was 151,706 bp, and the mitochondria was 304,060 bp. We then used these assembled sequences as bait to remove from the genome assembly all contigs/scaffolds that represented fragments of the chloroplast or mitochondria (i.e. the contigs/scaffolds were at least 99% identical to the assembled chloroplast or mitochondria over at least 99% of their total length). Adding the assembled chloroplast and mitochondria genomes to the remaining sequences resulted in a final epazote genome assembly containing 191 sequences (16 chromosome-scale scaffolds, the chloroplast and mitochondria, and 173 unincorporated contigs/scaffolds) spanning 469.23 Mbp (Table 1). Assessment of genome completeness using BUSCO identified 99.01% (1,598) of the 1,614 universal, single-copy orthologs in the embryophyta dataset in the epazote genome assembly, of which 88.17% (1,423) were single-copy and 10.84% (175) were duplicated (see Supplementary Table 1).

We annotated repeats using RepeatModeler and RepeatMasker and found that 51.81% (243.12 Mbp) of the genome assembly was categorized as repetitive sequence (see Supplementary Table 2). Repeat density was highest in the proximal regions of the chromosomes (Fig. 1). We also identified telomeric repeats in the genome assembly and found that they were present at both distal ends of 14 of the chromosomes and at 1 distal end of the remaining two chromosomes (DaChr9 and DaChr14; Fig. 1).

To annotate genes, we first performed PacBio Iso-Seq of pooled RNA samples from leaves, meristems, stems, and roots and assembled the reads into 209,676 transcripts. We used these transcripts as evidence—together with the OrthoDB.v11.viridiplantae, uniprotkb_Caryophyllales, and ncbi.Caryophyllales.refseq2 protein databases—to annotate 24,424 genes and 27,482 proteins using Braker3. Gene density patterns contrasted with repeat density, with the highest gene density observed at the distal ends of the chromosomes (Fig. 1). Assessment of completeness of the predicted proteins using BUSCO identified 97.33% (1,571) of the 1,614 universal, single-copy orthologs in the embryophyta dataset, of which 69.45% (1,121) were single-copy and 27.88% (450) were duplicated (see Supplementary Table 1).

**Fig. 2.**
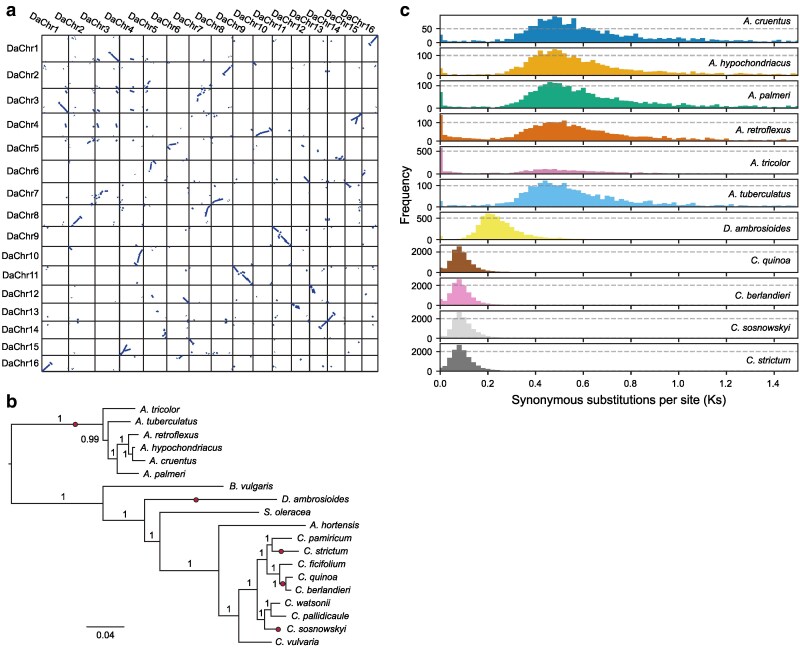
Collinearity and polyploidization in the epazote genome. a) Dot plot of homologous genes within the epazote genome. b) Phylogenetic relationships between epazote (*D. ambrosioides*) and several related species in the Amaranthaceae family. Circles indicate lineages experiencing a WGD. c) Histograms showing the distribution of Ks values for homologous gene pairs identified within epazote and several *Amaranthus* and *Chenopodium* species.

We identified homologous genes within the tetraploid epazote genome and plotted their positions to identify syntenic relationships among the chromosomes. A total of 12,525 collinear genes were identified (45.87% of the total genes). Although distinct regions of collinearity were observed, only 1 pair of likely homoeologous chromosomes could be identified, with DaChr9 and DaChr11 showing long stretches of collinear genes over almost the entirety of each chromosome (Fig. 2a). A small number of chromosomes show a 1:2 relationship. For example, approximately half of DaChr1 is collinear with half of DaChr3, and the other half of DaChr1 is collinear with half of DaChr16. The other halves of DaChr3 and DaChr16 show only small stretches of collinear genes with several other chromosomes. A similar pattern is observed between DaChr8 aligning with DaChr2 and DaChr7. Together, these results suggest that epazote is a relatively old tetraploid that has undergone substantial pseudogenization and rediploidization, which is also reflected in the relatively low number of intact duplicate genes as assessed via BUSCO.

To test this hypothesis, we compared the distribution of synonymous substitutions per synonymous site (Ks) values for homologous gene pairs in epazote and several other polyploid species in the Amaranthaceae family. A single WGD is shared among several *Amaranthus* species, whereas multiple independent WGDs have occurred in *Chenopodium* (Fig. 2b). We found that the WGD we observed in epazote is independent from those in *Amaranthus* and *Chenopodium* (Fig. 2c). Analysis of the distribution of Ks values for homologous gene pairs suggests that the epazote WGD might be younger than the WGD in *Amaranthus* and older than the WGDs in *Chenopodium*, which are all roughly the same age. This is supported by the more extensive collinearity observed within the epazote genome than within the amaranth genome (Lightfoot et al. 2017), but less than that observed within the quinoa genome (Rey et al. 2023).

To provide additional genomic resources in the genus, we performed whole-genome resequencing of 3 additional accessions of *D. ambrosioides*, 5 accessions of *D. graveolens*, and 1 accession each of *D. anthelmintica*, *D. botrys*, and *D.* Cristata (Table 2). We mapped sequencing reads from each accession onto the reference epazote genome assembly and observed dramatic differences in the percentage of reads that successfully mapped to the reference (Table 2). For example, as would be expected, the highest read mapping percentage was observed in the other accessions of *D. ambrosioides*, with all 3 showing greater than 98% mapping. A high percentage of reads (84.45) also mapped for *D. anthelmintica*, which has been shown to be closely related to *D. ambrosioides* (Uotila et al. 2021). The remaining resequenced species are known to be much more distantly related to *D. ambrosioides*, and this is confirmed by the low percentage of reads (all between 30% and 40%) that mapped to the epazote reference genome. *Dysphania* is known to contain species of varying ploidy levels (Uotila et al. 2021), and the stark differences in read mapping percentages that we observed could also indicate different ploidy levels among the resequenced accessions.

**Table 2.** Accessions used for resequencing.

Accession	Species	Origin	Raw data (Gbp)	Reads mapped to reference (%)
PI 612852	*Dysphania ambrosioides*	AZ, USA	6.67	98.90
PI 612853	*Dysphania ambrosioides*	AZ, USA	10.76	98.37
PI 686469	*Dysphania ambrosioides*	FL, USA	6.31	99.59
PI 689078	*Dysphania anthelmintica*	FL, USA	6.92	84.45
PI 605702	*Dysphania botrys*	Poland	6.26	38.25
BYU804	*Dysphania cristata*	Australia	6.53	32.58
Dg0728	*Dysphania graveolens*	CO, USA	7.11	31.77
BYU468	*Dysphania graveolens*	AZ, USA	7.10	39.64
PI 674270	*Dysphania graveolens*	NM, USA	6.86	42.61
W6 46807	*Dysphania graveolens*	NM, USA	6.87	43.10
W6 46809	*Dysphania graveolens*	NM, USA	6.65	42.69

In summary, we present a new, highly complete genome assembly and annotation for epazote (*D. ambrosioides*) and add additional evidence to the history of polyploidization within Amaranthaceae forming a foundation for future work into the biology of this diverse family of plants.

## Supplementary Material

jkaf191_Supplementary_Data

## Data Availability

The genome assembly and raw sequencing data are available on NCBI under BioProject accession number PRJNA821252. The genome assembly fasta file and annotation gff file can also be downloaded from https://quinoadb.org/download/Dysphania/Dysphania_ambrosioides/. All scripts used are available at https://github.com/hubjarvis/epazote_genome/blob/main/README. Supplemental material available at G3 online.
