# Supplementary material for: A chromosome-scale genome assembly and annotation of the tetraploid herb “epazote” (Dysphania ambrosioides)
Source: G3 (Bethesda). 2025 Aug 19;15(11):jkaf191. doi: 10.1093/g3journal/jkaf191 (PMC12608073; doi:10.1093/g3journal/jkaf191)
Supplement: jkaf191_Supplementary_Data [file jkaf191_supplementary_data.pdf]

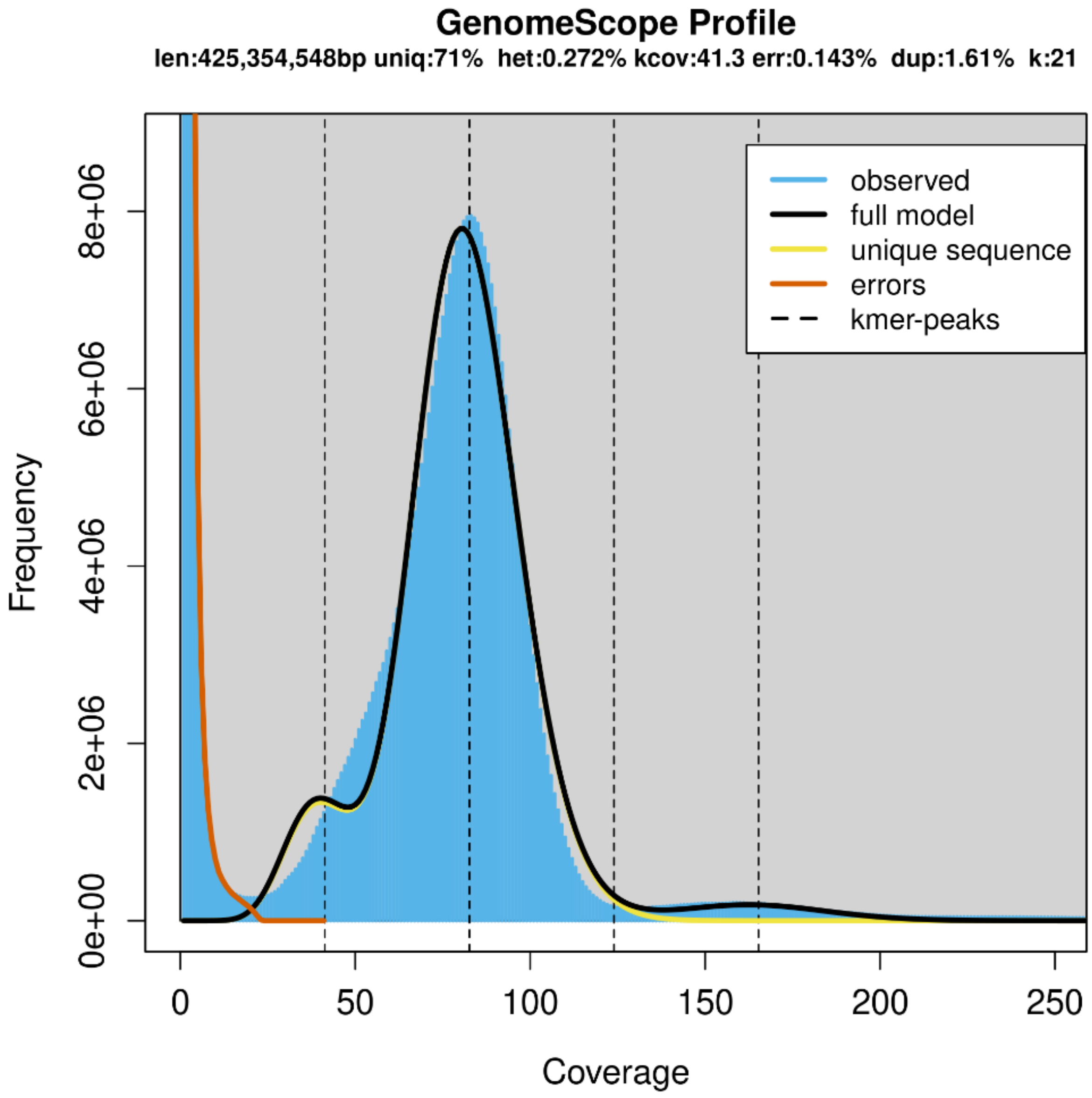


**Supplementary Figure 1.** Estimate of genome size using kmer frequency analysis implemented in GenomeScope.

a

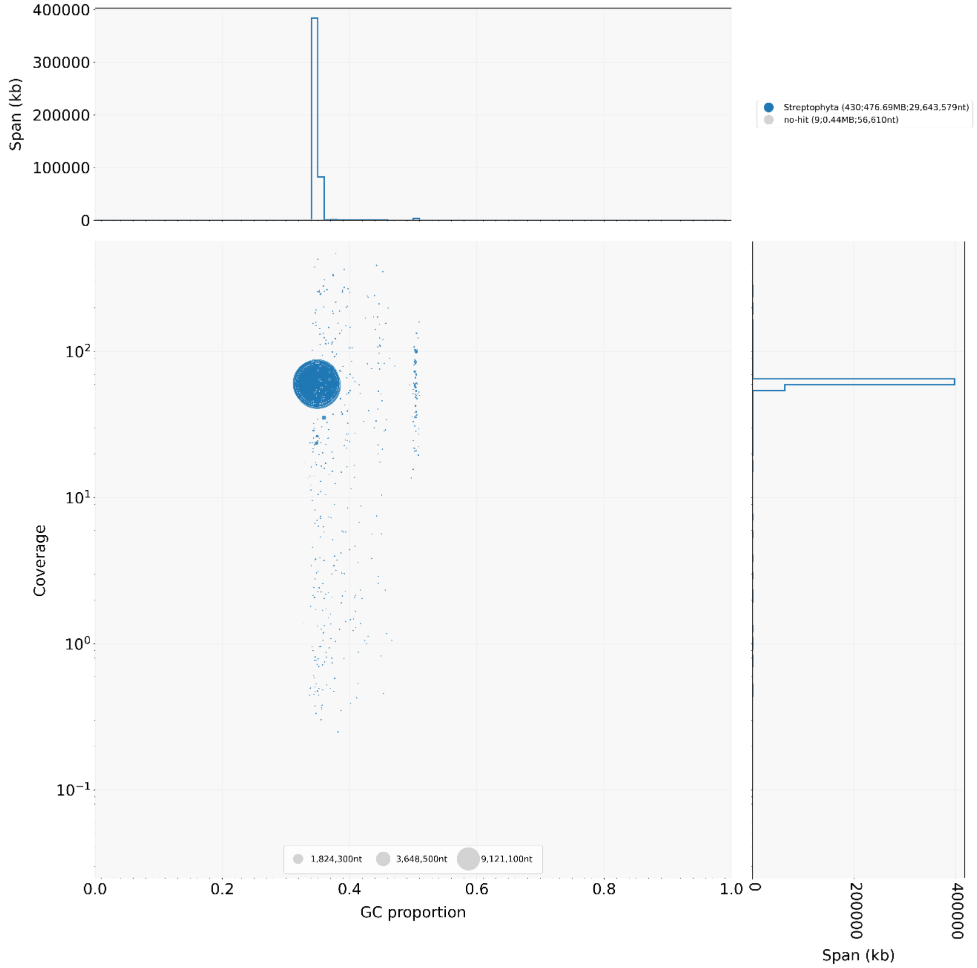


b

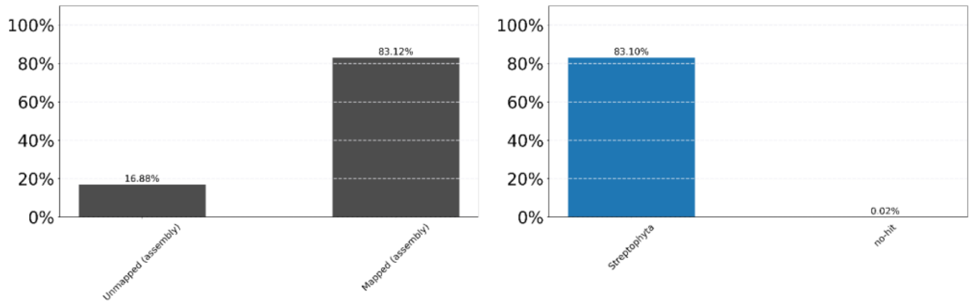


**Supplementary Figure 2.** BlobTools assessment of putative contaminant sequences in the epazote genome based on coverage and GC proportion (a) or read mapping and sequence homology (b).

**Supplementary Table 1.** Assessment of completeness of the epazote genome and annotated proteins using BUSCO.

| Universal, single-copy orthologs | Genome | Proteins |
|---|---|---|
| Complete, single-copy | 1,423 (88.17%) | 1,121 (69.45%) |
| Complete, duplicated | 175 (10.84%) | 450 (27.88%) |
| Fragmented | 7 (0.43%) | 4 (0.25%) |
| Missing | 9 (0.56%) | 39 (2.42%) |

**Supplementary Table 2.** Repetitive sequences identified in the epazote genome using RepeatModeler and RepeatMasker.

| Class | Count | Length (bp) | Percentage |
|---|---|---|---|
| DNA | 2,204 | 847,575 | 0.18 |
| CMC-EnSpm | 11,075 | 8,722,329 | 1.86 |
| Dada | 205 | 246,743 | 0.05 |
| Ginger | 1,207 | 129,187 | 0.03 |
| MULE-MuDR | 2,952 | 1,448,705 | 0.31 |
| MuLE-MuDR | 8,145 | 6,366,373 | 1.36 |
| PIF-Harbinger | 1,889 | 930,543 | 0.20 |
| Sola | 532 | 53,326 | 0.01 |
| TcMar-Mogwai | 702 | 510,228 | 0.11 |
| TcMar-Stowaway | 18,691 | 3,869,513 | 0.82 |
| hAT | 2,793 | 345,275 | 0.07 |
| hAT-Ac | 16,495 | 6,806,866 | 1.45 |
| hAT-Charlie | 344 | 103,970 | 0.02 |
| hAT-Tag1 | 2,582 | 406,241 | 0.09 |
| hAT-Tip100 | 2,182 | 688,501 | 0.15 |
| LINE | | | |
| CRE-II | 69 | 81,691 | 0.02 |
| L1 | 5,602 | 3,864,926 | 0.82 |
| R2 | 288 | 69,651 | 0.01 |
| RTE-BovB | 4,413 | 1,356,468 | 0.29 |
| LTR | 983 | 207,475 | 0.04 |
| Caulimovirus | 614 | 1,034,755 | 0.22 |
| Copia | 26,394 | 35,138,701 | 7.49 |
| Gypsy | 36,287 | 45,699,990 | 9.74 |
| Pao | 498 | 151,662 | 0.03 |
| RC | | | |
| Helitron | 983 | 694,148 | 0.15 |
| SINE | | | |
| tRNA-RTE | 266 | 29,150 | 0.01 |
| Unknown | 237,792 | 109,551,081 | 23.35 |
| Low_complexity | 13,700 | 678,646 | 0.14 |
| Satellite | 124 | 61,333 | 0.01 |
| Simple_repeat | 102,874 | 8,894,292 | 1.90 |
| rRNA | 496 | 4,129,753 | 0.88 |
| Total | 503,381 | 243,119,097 | 51.81 |
